# Data Science: Measuring Uncertainties

**DOI:** 10.3390/e22121438

**Published:** 2020-12-20

**Authors:** Carlos Alberto de Braganca Pereira, Adriano Polpo, Agatha Sacramento Rodrigues

**Affiliations:** 1 Department of Statistics, Institute of Mathematics and Statistics, University of Sao Paulo, Sao Paulo 05508-090, Brazil; 2 Department of Mathematics and Statistics, The University of Western Australia, Perth, WA 6009, Australia; adriano.polpo@uwa.edu.au; 3 Department of Statistics, Federal University of Espirito Santo, Vitoria 29075-910, Brazil; agatha.rodrigues@ufes.br

With the increase in data processing and storage capacity, a large amount of data is available. Data without analysis does not have much value. Thus, the demand for data analysis is increasing daily, and the consequence is the appearance of a large number of jobs and published articles in this area.

Data science has emerged as a multidisciplinary field to support data-driven activities, integrating and developing ideas, methods and processes to extract information from data. There are methods built from different areas of knowledge: Statistics, Computer Science, Mathematics, Physics, Information Science and Engineering, among others. This mixture of areas gave rise to what we call Data Science.

New solutions to new problems have been proposed rapidly for large volumes of data. Current and future challenges require greater care in creating new solutions that satisfy the rationality of each type of problem. Labels such as Big Data, Data Science, Machine Learning, Statistical Learning and Artificial Intelligence are demanding more sophistication in their foundations and in the way they are being applied. This point highlights the importance of building the foundations of Data Science.

This Special Issue is dedicated to solutions and discussions of measuring uncertainties in data analysis problems. The twelve articles in this edition discuss data science problems. The articles consider the reasoning behind their proposed solutions and illustrate how to apply them either in a real dataset or simulated dataset.

As stated earlier, multidisciplinarity is an important feature of data science, and this is clearly presented in this Special Issue. Ref. [1] proposes a new method for modelling problems and a data-clustering framework, and ref. [2] considers the estimation of the probability density function. In terms of the stochastic process, ref. [3] considers the fundamental properties of Tensor Markov Fields. Under a Bayesian paradigm of Statistical Inference, ref. [4] proposes a solution to classification problems.

Time series is one of the most prominent areas in data science, and some of the articles published here propose solutions with practical motivations in this area [5,6,7,8]. As mentioned before, this Special Issue encouraged articles on the foundations of measuring uncertainty [9,10,11,12].

The first article of this Special Issue was published on 30 October 2019, and the last on 26 October 2020. The articles are briefly discussed below, in order of the date of submission.

Due to its flexibility for treating heterogeneous populations, mixture models have been increasingly considered in modelling problems, and it provides a better cluster interpretation under a data-clustering framework [13].

In the traditional literature solutions, the results of the mixture model fit are highly dependent on the choice of the number of components fixed a priori. Thus, selecting an incorrect number of mixture components may cause the non-convergence of the algorithm and/or a short exploration of the clusterings [1].

Ref. [1] is the first published article in this issue. The authors propose an integrated approach that jointly selects the number of clusters and estimates the parameters of interest, without needing to specify (fix) the number of components. The authors developed the ISEM (integrated stochastic expectation maximisation) algorithm where the allocation probabilities depend on the number of clusters, and they are independent of the number of components of the mixture model.

In addition to theoretical development and evaluation of the proposed algorithm through simulation studies, the authors analyse two datasets. The first one refers to velocity in km/s of 82 galaxies from 6 well-separated conic sections of an unfilled survey of the Corona Borealis region; this is well-known Galaxy data in the literature. The second dataset refers to an acidity index measured in a sample of 155 lakes in central-north Wisconsin.

By considering the estimation of the probability density function (pdf), ref. [2] presented a wide range of applications for pdf estimation are provided, exemplifying its ubiquitous importance in data analysis. They discuss the need for developing universal measures to quantify error and uncertainties to enable comparisons across distribution classes, by establishing a robust distribution-free method to make estimates rapidly while quantifying the error of an estimate.

The authors consider a high-throughput, non-parametric maximum entropy method that employs a log-likelihood scoring function to characterise uncertainty in trial probability density estimates through a scaled quantile residual (SQR). This work is based on [14]. The SQR for the true probability density has universal sample size invariant properties equivalent to the sampled uniform random data (SURD).

Several alternative scoring functions that use SQR were considered, and they compared the sensitivity in quantifying the quality of a pdf estimate. The scoring function must exhibit distribution-free and sample size invariant properties so that it can be applied to any random sample of a continuous random variable. It is worth noting that all the scoring functions presented in the article exhibit desirable properties with similar or greater efficacy than the Anderson Darling scoring function and all are useful for assessing the quality of density estimates.

They present a numerical study to explore different types of measures for SQR quality. The initial emphasis was on constructing sensitive quality measures that are universal and sample size invariant. These scoring functions based on SQR properties can be applied to quantifying the “goodness of fit” of a pdf estimate created by any methodology, without knowledge of the true pdf.

The scoring function effectiveness is evaluated using receiver operator characteristics (ROC) to identify the most discriminating scoring function, by comparing overall performance characteristics during density estimation across a diverse test set of known probability distributions.

Integer-valued time series are relevant to many fields of knowledge, and an extensive number of models has been proposed, such as the first-order integer-valued autoregressive (INAR(1)) model. Ref. [5] considered a hierarchical Bayesian version of the INAR(p) model with variable innovation rates clustered according to a Pitman–Yor process placed at the top of the model hierarchy.

Using the full conditional distributions of the innovation rates, they inspected the behaviour of the model as concentrating or spreading the mass of the Pitman–Yor base measure. Then, they presented a graphical criterion that identified an elbow in the posterior expectation of the number of clusters as varying the hyperparameters of the base measure. The authors investigated the prior sensitivity and found ways to control the hyperparameters in order to achieve robust results. A significant contribution is a graphical criterion, which guides the specification of the hyperparameters of the Pitman–Yor process base measure.

Besides the theoretical development, the proposed graphical criterion was evaluated in simulated data. Considering a time series of yearly worldwide earthquakes events of substantial magnitude (equal or greater than 7 points on the Richter scale) from 1900 to 2018, they compared the forecasting performance of their model against the original INAR(p) model. Ref. [6] considered the problem of model fit and model forecasting in time series. For that, the authors considered the singular spectrum analysis (SSA), that is a powerful non-parametric technique to decompose the original time series into a set of components that can be interpreted, such as trend components, seasonal components, and noise components. They proposed a robust SSA algorithm by replacing the standard least-squares singular value decomposition (SVD) by a robust SVD algorithm based on the L1 norm and a robust SSA algorithm. The robust SVD was based on the Huber function. Then, a forecasting strategy was presented for the robust SSA algorithms, based on the linear recurrent SSA forecasting algorithm.

Considering a simulation example and time-series data from investment funds, the algorithms were compared to other versions of the SSA algorithm and classical ARIMA. The comparisons considered the computational time and the accuracy for model fit and model forecast. Ref. [9] presented a discussion about hypothetical judgment and measures to evaluate that, and exemplified it using a diagnostic of the infection of the Coronavirus Disease (COVID-19). Their purposes are (1) to distinguish channel confirmation measures that are compatible with the likelihood ratio and prediction confirmation measures that can be used to assess probability predictions, and (2) to use a prediction confirmation measure to eliminate the Raven Paradox and to explain that confirmation and falsification may be compatible.

They consider the measure *F*, that is one of few confirmation measures which possess the desirable properties as identified by many authors: symmetries and asymmetries, normalisation, and monotonicity. Also, the measure b∗, the degree of belief, was considered and optimised with a sampling distribution seen as a confirmation measure, which is similar to the measure *F* and also possesses the above-mentioned desirable properties.

From the diagnosis of the infection of the COVID-19, they show that only measures that are functions of the likelihood ratio, such as *F* and b∗, can help to diagnose the infection or choose a better result that can be accepted by the medical society. However, measures *F* and b∗ do not reflect the probability of the infection. Furthermore, using *F* or b∗ is still difficult to eliminate the Raven Paradox.

The measures *F* and b∗ indicate how good a hypothesis test of means is compared to the probability predictions. Hence, the authors proposed a measure c∗ that can indicate how good a probability prediction is. c∗ is called the prediction confirmation measure and b∗ is the channel confirmation measure. The measure c∗ accords to the Nicod criterion and undermines the Equivalence Condition, and hence can be used to eliminate the Raven Paradox. Ref. [3] presented the definitions and properties of Tensor Markov Fields (random Markov fields over tensor spaces). The author shows that tensor Markov fields are indeed Gibbs fields whenever strictly positive probability measures are considered. It is also proved how this class of Markov fields can be built based on statistical dependency structures inferred on information-theoretical grounds over empirical data. The author discusses how the Tensor Markov Fields described in the article can be useful for mathematical modelling and data analysis due to their intrinsic simplicity and generality. Ref. [4] proposed a variational approximation on probit regression models with intrinsic priors to deal with a classification problem. Some of the authors’ motivations to combine intrinsic prior methodology and variational inference are to automatically generate a family of non-informative priors; to apply intrinsic priors on inference problems; intrinsic priors have flat tails that prevent finite sample inconsistency; for inference problems with a large dataset, variational approximation methods are much faster than MCMC-based methods.

The proposed method is applied to the LendingClub dataset (https://www.lendingclub.com). The LendingClub is a peer-to-peer lending platform that enables borrowers to create unsecured personal loans between $1000 and $40,000. Investors can search and browse the loan listings on the LendingClub website and select loans that they want to invest in. In addition, the information about the borrower, amount of loan, loan grade, and loan purpose was provided to them. The variable loan status (paid-off or charged-off) is the target variable, and [4] considers a set of predictive covariates, as loan term in months, employment length in years, annual income, among others. [10] constructed a decision-making model based on intuitionistic fuzzy cross-entropy and a comprehensive grey correlation analysis algorithm. Their motivation is the fact that despite the fact that intuitionistic fuzzy distance measurement is an effective method to study multi-attribute emergency decision-making (MAEDM) problems, the traditional intuitionistic fuzzy distance measurement method cannot accurately reflect the difference between membership and non-membership data, where it is easy to cause information confusion.

The intuitionistic fuzzy cross-entropy distance measurement method was introduced, which can not only retain the integrity of decision information but also directly reflect the differences between intuitionistic fuzzy data. Focusing on the weight problem in MAEDM, the authors analysed and compared the known and unknown attribute weights, which significantly improved the reliability and stability of decision-making results. The intuitionistic fuzzy cross-entropy and grey correlation analysis algorithm were introduced into the emergency decision-making problems such as the location ranking of shelters in earthquake disaster areas, which significantly reduced the risk of decision-making. The validity of the proposed method was verified by comparing the traditional intuitionistic fuzzy distance to the intuitionistic fuzzy cross-entropy.

The authors highlight that the proposed method applies to emergency decision-making problems with certain subjective preference. In addition to the theoretical approach and highlighting the importance to deal with disasters motivations, the authors took the Wenchuan Earthquake on May 12th 2008 as a case of study, constructing and solving the ranking problem of shelters.

Motivated by time series problems, ref. [7] reviewed the shortcomings of unit root and cointegration tests. They proposed a Bayesian approach based on the Full Bayesian Significance Test (FBST), a procedure designed to test a sharp or precise hypothesis.

The importance of studying this is justified by the fact that one should be able to assess if a time series present deterministic or stochastic trends to perform statistical inference. For univariate analysis, one way to detect stochastic trends is to test if the series has unit-roots (unit root tests). For multivariate studies, determining stationary linear relationships between the series, or if they cointegrate (cointegration tests) are important.

The Augmented Dickey–Fuller test is one of the most popular tests used to assess if a time series has a stochastic trend or if they have a unit root for series described by auto-regressive models. When one is searching for long-term relationships between multiple series, it is crucial to know if there are stationary linear combinations of these series, i.e., if the series are cointegrated. One of the most used tests is the maximum eigenvalue test.

Besides proposing the method considering FBST, the authors also compared its performance with the most used frequentist alternatives. They have shown that the FBST works considerably well even when one uses improper priors, a choice that may preclude the derivation of Bayes Factors, a standard Bayesian procedure in hypotheses testing. Ref. [11] considered a Kalman filter and a Rényi entropy. The Rényi entropy was employed to measure the uncertainty of the multivariate Gaussian probability density function. The authors proposed calculation of the temporal derivative of the Rényi entropy of the Kalman filter’s mean square error matrix, which provided the optimal recursive solution mathematically and was minimised to obtain the Kalman filter gain.

One of the findings of this manuscript was that, from the physical point of view, the continuous Kalman filter approached a steady state when the temporal derivative of the Rényi entropy was equal to zero, which means that the Rényi entropy remained stable.

A numerical experiment of falling body tracking in noisy conditions with radar using the unscented Kalman filter, and a practical experiment of loosely-coupled integration, are provided to demonstrate the effectiveness of the above statements and to show the Rényi entropy truly stays stable when the system becomes steady.

The knowledge about future values and the stock market trend has attracted the attention of researchers, investors, financial experts, and brokers. Ref. [8] proposed a stock trend prediction model by utilising a combination of the cloud model, Heikin–Ashi candlesticks, and fuzzy time series in a unified model.

By incorporating probability and fuzzy set theories, the cloud model can aid the required transformation between the qualitative concepts and quantitative data. The degree of certainty associated with candlestick patterns can be calculated through repeated assessments by employing the normal cloud model. The hybrid weighting method comprising the fuzzy time series, and Heikin–Ashi candlestick was employed for determining the weights of the indicators in the multi-criteria decision-making process. The cloud model constructs fuzzy membership functions to deal effectively with uncertainty and vagueness of the historical stock data to predict the next open, high, low, and close prices for the stock.

The objective of the proposed model is to handle qualitative forecasting and not quantitative only. The experimental results prove the feasibility and high forecasting accuracy of the constructed model. Ref. [12] uses the maximum entropy principle to provide an equation to calculate the Lagrange multipliers. Accordingly, an equation was developed to predict the bank profile shape of threshold channels.

The relation between ratio with the entropy parameter and the hydraulic and geometric characteristics of channels was evaluated. The Entropy-based Design Model of Threshold Channels (EDMTC) for estimating the shape of banks profiles and the channel dimensions was designed based on the maximum entropy principle in combination with the Gene Expression Programming regression model.

The results indicate that the entropy model is capable of predicting the bank profile shape trend with acceptable error. The proposed EDMTC can be used in threshold channel design and for cases when the channel characteristics are unknown.

It is our understanding that this Special Issue contributes to increasing knowledge in the data science field, by fostering discussions of measuring uncertainties in data analysis problems. The discussion of foundations/theoretical aspects of the methods is essential to avoid the use of black-box procedures, as well as the presentation of the methods in real problem data. Theory and application are both important to the development of data science.
